# Student-led clinic cervical cancer screening—medical students’ views on progression of learning, quality of Pap smears and women´s experiences of the visit – a mixed methods study

**DOI:** 10.1186/s12909-023-04162-y

**Published:** 2023-04-05

**Authors:** Caroline Lilliecreutz, Anna Clara Spetz Holm, Madeleine Abrandt Dahlgren, Marie Blomberg

**Affiliations:** 1 Department of Biomedical and Clinical Sciences, Linköping University, Linköping, Sweden; 2 Department of Obstetrics and Gynecology, Linköping University, Linköping, Sweden; 3 Department of Medicine and Health Sciences, Linköping University, Linköping, Sweden

**Keywords:** Student-led clinic cervical cancer screening (SLC-CCS), Medical students, Gynecology, Papanicolaou smear

## Abstract

**Background:**

Student-led clinics (SLC) have been described, but not in gynecology. Gynecology is a subject typically covered in the last terms of medical training, however it includes few opportunities for students to tackle all phases of a consultation and a shortage of opportunities to perform gynecological examinations. Therefore, we started a student-led clinic for cervical cancer screening (SLC-CCS) in Linköping, Sweden and aimed to evaluate students’ views on the progression of learning, the quality of the Papanicolaou (Pap) smear, and women´s experiences of the visit, using mixed methodology.

**Methods:**

The implementation of the SLC-CCS is described in detail. Students (*n* = 61) taking part in the SLC-CCS between January and May 2021 were invited to participate in a follow-up discussion (*n* = 24) focused around four themes: attitudes and expectations prior to participation, experiences of the patient encounter, organization of the placement, and reflections on and suggestions for further development of the placements. The group meetings were conducted in Swedish, recorded, transcribed verbatim and subjected to a qualitative, descriptive thematic analysis. Thematic analysis is considered an appropriate method of analysis for seeking to understand experiences, thoughts, or behaviors across a data set. The proportion of Pap smears lacking cells from the squamous epithelium during the study period was compared with data from the same clinic before the SLC-CCS started. A validated questionnaire on women’s experience of the Pap smear visit was provided. Answers were compared between women who had the Pap smear taken by a student or a healthcare provider.

**Results:**

Three different themes were generated: *growing confidence in the clinical situation, embodied awareness of variation in anatomy, doubting accuracy of one’s own performance.* The percentage of Pap smears lacking cells from the squamous epithelium were equal (2%) during the study period compared to the period before the SLC-CCS started (*p* = 0.28). No difference was found in the satisfaction index between the women examined by a student, those examined by a healthcare provider, or women who did not know who the examiner was (*p* = 0.112).

**Conclusions:**

The students expressed a growing confidence in the clinical situation and there was high satisfaction from the women. The quality of the Pap smears taken by the students was equal to the quality of those taken by the health care staff. All these findings indicate that high patient safety was maintained during this activity support the recommendation to include SLC-CCS as part of the medical training.

**Supplementary Information:**

The online version contains supplementary material available at 10.1186/s12909-023-04162-y.

## Background

In Sweden, the subject of gynecology- covered in the last terms of medical training, is new and therefore often considered stimulating and interesting Almost five years have passed, and the students are soon about to leave medical school.. However, educational methodology is very similar to earlier terms. The students are professional listeners and observers. There is certainly lack of opportunities where students are expected to contribute to patient care, taking responsibility for the patient during the whole visit, and tackling all the phases of a patient consultation (e.g. professional treatment, information and answering questions). There is also a shortage of opportunities for the students to perform enough gynecological examinations to master this skill in their future professional life, for example in primary health care facilities [1]. The gynecological examination could be viewed as an entrustable professional activity (EPA), defined as clinically relevant, frequently occurring, and an examination that a physician should be able to achieve on his/her own from the first day of work [2].

The research team (also responsible for the education in gynecology on the medical program) scrutinized all available tasks in our specialty and pinpointed the cervical cancer screening program as an opportunity for development of a student-led clinic—cervical cancer screening (SLC-CCS) where the students can contribute to health care by working under the supervision of a midwife, nurse or a specially trained assistant nurse, and where they can perform many gynecological examinations. The cervical cancer screening program in Sweden at the time of this study was free of charge and offered to all women between the age 23 to 70 years. The coverage rate of the program was almost 80% (2021). The incidence of cervical cancer in Sweden is about 11/100000 and approximately 650 women are diagnosed with cervical cancer every year (data from the Swedish National Board of Health and Welfare).

Student-led clinics (SLCs), as a model for clinical placements have become more common in education for health professionals [3–5]. SLCs can be seen as a way to provide learners with authentic learning experiences while immersed in clinical practice [6]. A recent study of students’ and patients’ experiences of participation in a SLC caring for patients after a total hip arthroplasty by Niva & MacLellan (2021) showed that patients were satisfied with their care [6]. Students felt well-supported and developed confidence and independence, taking responsibility for the care provided. The authors suggest that SLCs can provide high quality experiential learning opportunities, and that they may offer pedagogical benefits compared to traditional clinical placements. Similar results were found in a study of patients’ and students’ experiences of participating in a SLC in oncology, where pairs of medical students were able to lead a consultation with a cancer patient to discuss radical and palliative therapies. Patients were highly satisfied, and students stated that the SLC provided an invaluable experience, and also increased their interest in oncology [7]. The SLC model has also been applied with successful results for medical students’ clinical placements in psychiatric outpatient care [8].

Nicole and coworkers presented a paper in 2016 including 12 tips and recommendations for establishing a hospital-based student-led service [9]. The paper identified some key features for a student-led service that supported our choice of the cervical cancer screening program. The care should be evidence-based and performed in a safe and supportive learning environment. Furthermore, the authors advocated outcomes that needed to be clearly defined, both student learning outcomes as well as evidence-based patient outcomes [9].

To the best of our knowledge, no previous studies have described the development of a SLC-CCS or have examined its impact on student learning, quality outcomes and the experiences of the attending women.

Therefore, the aims of the present study were to use a mixed methodology to evaluate the SLC-CCS in three dimensions: student´s views on the progression of learning, the quality of the Papanicolaou (Pap) smear, and women´s experiences of their visit.

## Methods

The present study applied a mixed methods approach, combining quantitative outcome data with qualitative analyses of interviews. Mixed methods research approaches are commonly occurring in the literature and studies can be designed in different ways, to make possible to combine findings from quantitative and qualitative research [10]. The rationale for a mixed method approach was that the quantitative data we gathered on students' performance would not adequately address any issues or dilemmas they faced concerning their learning in the situation. To understand what participating in the SLC-CCS as a learning experience meant to students, we decided to conduct focused discussion groups after participation in the clinic. This would enable us to integrate the findings and consider how the SLC-CCS could be subject to development and improvement to support students' learning. In this study, we used a convergent mixed method design, meaning that quantitative and qualitative data collection occurred in parallel. The data were analyzed separately and then merged to provide a deeper and broader understanding [11, 12].

### Cervical cancer screening program

At the time of the study, the cervical cancer screening program in Region Östergötland, Sweden, invited women 23–70 years old for a Pap smear at an interval of every three years when between the ages of 23–49, and every five years at the ages of 50–70 years. The Pap smear were taken with the SurePath Liquid-based PapTest. The primary analysis in the Pap smears was cytology and human papilloma virus (HPV) testing (type 16, 18, 31, 33, 35, 39, 45, 51, 52, 56, 58, 59, 66 and 68) in the two age groups, respectively. For some women with previous abnormal cytology or positive HPV, both cytology and HPV testing were carried out. A national goal to maintain quality and confirm that the Pap smear is taken from the cervix is to minimize the proportion of tests that lack cells from the squamous epithelium [13].

### Creation and implementation of the SLC-CCS

We started in October 2020 by putting together a steering committee with clear roles and competences that would lead the project. The steering committee included a professor in Obstetrics and Gynecology (MB), the lead consultant for the cervical cancer screening program (CL), the course coordinator for ob/gyn (ACSH), the process leader of cervical cancer screening, two dedicated medical students who would attend the ob/gyn course in spring 2021 and a professor in medical pedagogy (MAD). A theoretical and practical introduction for the students concerning the cervical cancer screening program was then created, planned and incorporated into their schedule in advance of their SLC-CCS (Additional file 1)The theoretical introduction lasted four hours and included a presentation of clinical guidelines for cervical cancer screening. In addition, students were referred to two informative training websites [14, 15] as part of their preparation for the SLC-CCS. These websites included detailed instructions and pictures showing how to take the PAP smear. Finally, students completed a mandatory online knowledge test that they had to pass [16]. Participation in the SLC-CSS was part of the students' compulsory training and not voluntary. All students participating in the SLC-CSS were in their fifth year of medical school.The practical introduction comprised doing the actual pap smears. The first two pap smears were taken in the presence of the supervisor (midwife, nurse, or assistant nurse). One or two half-day sessions were planned for each student and there were two students per session. The students worked in separate rooms. A detailed flowchart including all practical instructions for the day was constructed.

To inform the women attending the cervical cancer screening outpatient clinic, a sign on the wall in the waiting room declared that medical students (their forenames) would take the Pap smears on that day. The forename of the supervisor of the day was also announced on the same sign. All women had the opportunity to wait for the next provider if the first one, for any reason, didn’t fulfilled her requirements. No notes on numbers or reasons were performed.

If a student became ill or for some reason was unable to attend the SLC-CCS, the student had to arrange replacement by another student on the ongoing course. The only care offered to the women attending the clinic was to take a pap smear as part of the cervical cancer screening program in order to detect cervical cancer or dysplasia.

The two student members of the steering group were deeply committed to both planning the theoretical half day as well as organizing the practical work at the SLC-CCS. Furthermore, the plan of a the SLC-CCS was supported by the management team of the Department of Gynecology and Obstetrics and the head of the medical program at Linköping University. Finally, presentations including the aims, practical issues and the components of the planned evaluation were given for the staff involved in the cervical cancer screening outpatient clinic.

### Qualitative data collection

The students taking part in the SLC-CCS clinic were informed prior to the placement about the planned research project and gave their informed consent to participate in the study. A convenience sampling strategy was applied, in that all students were invited to participate in a focused follow-up discussion group after the placement. Students who accepted the invitation (*n* = 41) received a link to an online meeting, and 24 students, of which 14 were women and 10 were men, participated in the interviews. The focused discussion group interviews were conducted by one of the authors (MAD) through online meetings on nine occasions between March and June 2021. Meetings were scheduled the week after completion of the placement. Between one to four students took part in each session. The discussion was focused around students’ views on four topics; 1) attitudes and expectations prior to participation in the SLC-CCS, 2) experiences of the patient encounter, 3) organization of the placement, and 4) reflections on and suggestions for further development of the placements. The interview guide could be seen in Additional file 2. The meetings were conducted in Swedish, recorded (audio and video) and later transcribed verbatim. The transcriptions were subjected to a descriptive thematic analysis [17], focusing on identifying common themes across the interviewed groups of students. The analysis involves a six steps process; 1) familiarizing yourself with the data, 2) generating initial codes, 3) searching for themes, 4) reviewing themes, 5) defining and naming themes [18]. Quotations from the focus group discussions that were illustrating the identified themes were selected and later translated into English. The preliminary findings were discussed between the whole research team.

### Quantitative data collection and statistics

The cytologists at the pathology department registered all Pap smear tests from the cervical screening program, including any that lacked cells from the squamous epithelium in the Pap smear test. The data were stored in a local database and extracted anonymously. The proportion of Pap smear tests lacking cells from the squamous epithelium during the study period (1st of February 2021 until the 27th of May 2021) was compared with data from the same clinic before the SLC-CCS started (1st of August 2020 until the 31st of December 2020).

A questionnaire to examine the women’s experience of thevisit at the SLC-CCS was used (Additional file 3) The questionnaire was created by a group of midwives and physicians from the Swedish national cervical screening group in 2013 with the intention of developing a national questionnaire. The questionnaire was tested in focus groups and then approved. The questionnaire was only available in Swedish (although translated for the purpose of the present study), and consisted of 11 questions concerning age, mother tongue, highest completed education, self-assessed general health, and questions related to experiences of the visit. The answers were in multiple choice format. The women also stated which occupational category who carried out the Pap smear test, i.e.. midwife, nurse, assistant nurse or a medical student. If they did not know who carried out the test, that was also registered. After the women had attended a Pap smear visit they received a paper with a quick response (QR) code from the person who carried out the test or they- could scan the QR code which was available on a poster in the waiting room. They could thereby download the questionnaire after the visit and answer the questions. The questionnaire was provided from 4^th^ of April until 17^th^ of June 2021. It took approximately three minutes to complete the questionnaire. Answers from the women were then compared between three groups; a medical student took the Pap smear group, a healthcare provider took the Pap smear group and a “don’t know who took the Pap smear” group.

Continuous data is presented as mean and standard deviation. Categorical data is presented as number and per cent. Categorical data was compared using Pearson’s Chi-square statistics and when appropriate Fischer’s exact test. Statistical significance was set to a *p*-value < 0.05 (two-sided).

## Results

### Qualitative data

The analyses were conducted by one of the authors with expertise knowledge in qualitative educational research within health professions education (MAD). The findings portray students’ perspectives on how the clinical placement at the SLC-CCS supported their learning. Three different themes were generated, illustrating reflections on experienced achievements as well as experienced worries or shortcomings.

The themes were: *growing confidence in the clinical situation, embodied awareness of variation in anatomy, doubting the accuracy of one’s own performance.*

#### Growing confidence in the clinical situation

A common theme, repeatedly found across all discussions, was the experience of a growing confidence in handling both the encounters with the women as well as the gynecological examination and procedure. The experiences of authenticity and responsibility for the situation were pointed out as important contributors to the growing confidence. One of the participants in FG3 expressed this as.

*M:1 It was like… even if there were staff in charge there, they were dependent on us, if one of us was sick, another student needed to step in as a replacement… So it was like being a member of the ordinary staff, and contributed to the system in that way.(FG3).*

The feeling of being part of the team in charge, and the number of patient encounters and examinations together led to students becoming comfortable in an otherwise often awkward and sensitive situation that medical students seldom get to practice. The following excerpts from the focused discussions (FG1 and FG 2) illustrate the theme.

*W1: I think that the biggest was… to get all that practical training… (my colleague) and I saw, like 50 patients that morning, and it was really good to get the technique, and most of all, the handling of patient encounters, to feel comfortable in that situation.(FG1).*

*M1: I agree, examination technique and patient encounters, that was what you took with you the most… Personally, I think I made enormous progress between the first patient and the last one, that was a really rewarding feeling (FG1).*

#### Embodied awareness of variation in anatomy

A second theme repeatedly occurring across the discussions was the importance of experiencing the normal variation in anatomy. The many repeated gynecological examinations the students carried out gave them an embodied awareness and learning experience that each human body is unique. The value of building up a reference bank for the variation in normal anatomy, to learn to locate different anatomical structures and to be sensitive to how it feels to do this were aspects that were emphasized in the focused discussions.

*Kv:1 Everybody looks different, in different ways… and I have of course not learned all the different ways, but at least I have achieved a larger bank of images, or how to put it, for how people look…(FG2).*

*M: I did not have a lot of experience of gynecological exams prior to this… so to build up a reference of the normal anatomy and how it can vary, meant a lot…That, in its own right, made the examinations easier and easier, and made you more secure in the encounter with the patient; you dared to make decisions, you learned to recognize how uncomfortable you were, or if you needed to call for the supervisor, or if you felt you could handle it yourself (FG1).*

#### Doubting the accuracy of one’s own performance

The third theme, discerned in some of the discussions, is one of self-criticism. Even if students felt their confidence growing and their capacity for handling encounters and examination of patients expanding, there were also expressions of an overall doubt in the accuracy of their own performance, since there was no immediate feedback on whether the sample was correctly taken or not.

*W:2…and afterwards… you are wondering… did I get good samples from everyone… or did I not get any good samples…?Well, you wonder, even if you felt rather satisfied then, when you had taken the pap smear, you still did not know if it was correct…* FG2.

### Quantitative data

A total number of 80 SLC-CCS were performed during the study period, which lasted from the 1st of February 2021 until the 31st of May 2021. Sixty-one medical students had these SLC-CCS, and thus each student had on average 1.3 (80/61) receptions of SLC-CCS each.

### Quality of pap smears

On the above outpatient visits, 3617 Pap smears were taken by students or sometimes when the student needed it, with help from the attending staff. On average, 45 (3617/80) Pap smears were taken at each SLC-CCS. The quality of the Pap smears is presented in Table 1.

**Table 1:** Pap smear quality

	Cervical cancer screening The 1^st^ of August 2020 until the 31st of December 2020 *N* = 11,572 n (%)	Student-led clinic cervical cancer screening (SLC-CCS) The 1^st^ of February 2021 until the 31^st^ of May 2021 *N* = 3616 n (%)	*p*-value*
Pap smears with cells from the squamous epithelium	11,355 (98%)	3538 (98%)	0.28
Pap smears without cells from the squamous epithelium	217 (2%)	78 (2%)	

^*^chi^2^test

Only one Pap smear lacked enough material to be analyzed and 2.0% (*n* = 78/3616) lacked cells from the squamous epithelium. When data from the period lasting from the 1st of August 2020 until the 31st of December 2020 before the SLC-CCS started was evaluated, it was found that a total of 11,575 Pap smears were taken, three lacked enough material to be analyzed and 2.0% (*n* = 217/11572) lacked cells from the squamous epithelium. There was no difference between the two time periods concerning the number of Pap smears that lacked cells from the squamous epithelium (*p* = 0.28).

### Questionnaire

Questionnaires were provided to all women who attended a Pap smear visit at the outpatient clinic from 4^th^ of April until 17^th^ of June 2021, i.e., two months during the time of the SLC-CCS and afterwards. In total, 177 women answered the questionnaire, and all were eligible for analysis; 78 (44.1%) women answered that the Pap smear was carried out by a healthcare provider, 30 (16.9%) said that a medical student carried out the test, and 64 (36.2%) did not know who carried out the test. Five women (2.8%) stated that both a healthcare provider and a medical student carried out the test; these five questionnaires were added to the group of samples taken by students, i.e., in total 35 (19.8%) women who had responded to the questionnaire were examined by students. There were no differences in age, educational level, self-assessed health or in the number of women stating that Swedish was their mother tongue between the three groups (Table 2).

**Table 2:** Characteristics of the women who answered the questionnaire

	All *N* = 177	Medical student *n* = 35 (19.8%)	Healthcare provider *n* = 78 (44.1%)	Don´t know *n* = 64 (36.2%)
Age Years^a^	*n* = 171 37.6 ± 10.8	*n* = 33 33.8 ± 10.2	*n* = 77 39.9 ± 11.4	*n* = 64 36.7 ± 9.6
Mother tongue, Swedish	*n* = 173 (97.7%)	*n* = 34 (97.1%)	*n* = 75 (97.4%)	*n* = 57 (89.1%)
Highest completed education				
Compulsory school	*n* = 4 (2.3%)	*n* = 0	*n* = 2 (2.6%)	*n* = 2 (3.1%)
Upper secondary school	*n* = 66 (37.5%)	*n* = 14 (40.0%)	*n* = 35 (45.5%)	*n* = 17 (26.6%)
University	*n* = 106 (60.2%)	*n* = 21 (60.0%)	*n* = 40 (51.9%)	*n* = 45 (70.3%)
Self-assessment of general health				
Excellent/Very good/Good	*n* = 162 (91.5%)	*n* = 31 (88.6%)	*n* = 73 (93.6%)	*n* = 58 (90.6%)
Tolerable	*n* = 13 (7.3%)	*n* = 4 (11.4%)	*n* = 5 (6.4%)	*n* = 4 (6.3%)
Bad	*n* = 2 (1.1%)	*n* = 0	*n* = 0	*n* = 2 (3.1%)

Group refers to the person who carried out the Pap smear

^a^mean and standard deviation

Almost nine of ten women (*n* = 154, 88.5%) were satisfied with the information that was given before the examination by invitation letter, brochure, or the healthcare giver irrespective of who examined them.

**Fig. 1:**
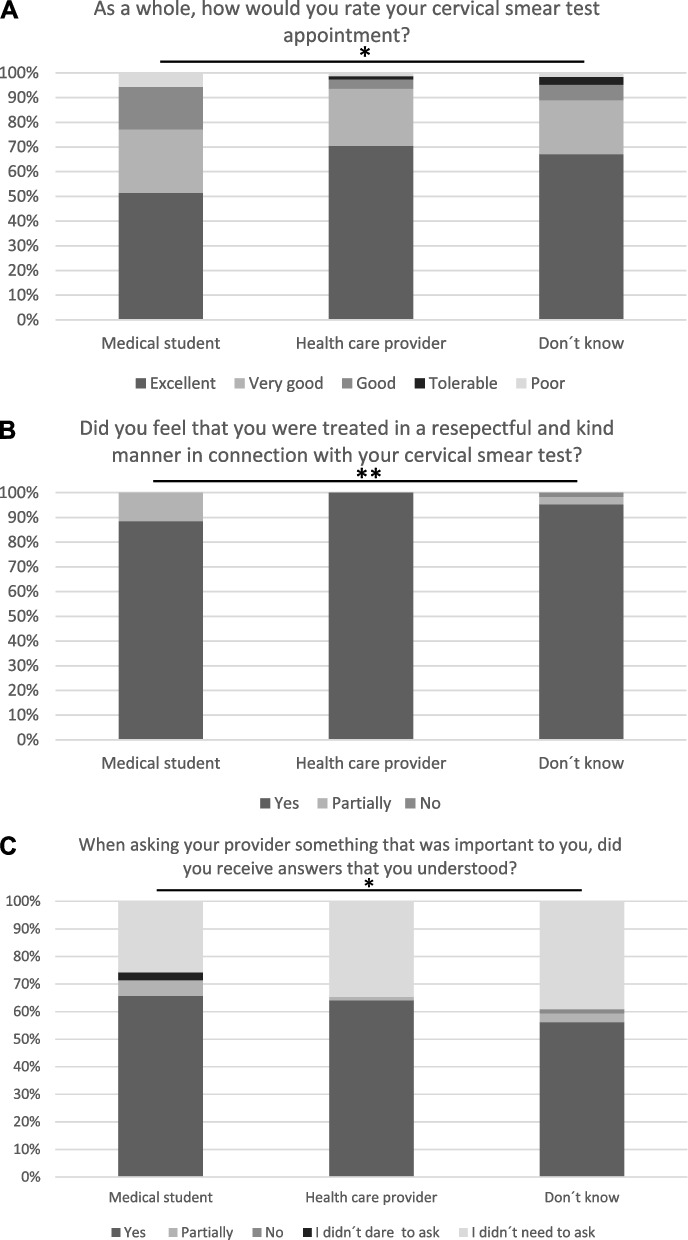
**A**-**C**. Women’s experiences of the visit at the SLC-CCS.Group refers to the person who carried out the Pap smear, *NS,** *p* = 0.005

There was a question concerning the experience of the visit as a whole when taking the Pap smear. The answers did not significantly differ among the examiner groups. However, in total, 96% (*n* = 170) of the women rated the experience of the visit as a whole as very good or good (Fig. 1A).

There was a question asking if the women were treated in a respectful and kind manner by the examiner. All women (*n* = 78) who were examined by healthcare providers reported that they were treated with total respect compared to 95.3% (*n* = 61) of women who did not know who the examiner was and 88.6% (*n* = 31) of the women examined by students (*p* = 0.005). Only one woman stated that she was not treated with respect, but she did not know who examined her (Fig. 1B).

Concerning questions to the examiner, two thirds of the women (*n* = 61; 34.5%) did not have any questions, and only one woman reported that she did not dare to ask. There were no significant differences concerning who the examiner was (*p* = 0.322).

In total, 115 women asked the examiner questions and 109 women (94.8%) stated that they were satisfied with the answer, irrespective of who the examiner was; 4.3% (*n* = 5) of the women who asked were partially satisfied with the answer; and only one woman (0.9%), who did not know who the examiner was, was not at all satisfied with the answer (Fig. 1C).

**Fig. 2:**
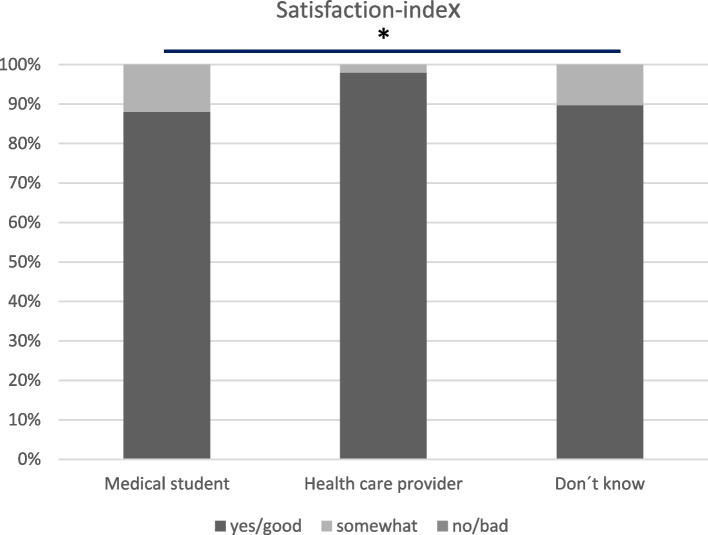
Women’s satisfaction with the visit at the SLC-CCS. Group refers to the person who carried out the Pap smear *NS

A “satisfaction index” was computed by combining the results from three questions (reported above separately in Fig. 1 A-C) in the questionnaires regarding the visit (Fig. 2).

At the visit, 115 women asked questions to the examiner. Since one of the questions in the “satisfaction index” were if they got answers on the questions that they understood, the results are based on these 115 questionnaires. We found no differences in the “satisfaction index” between the different groups of women examined by a medical student, a healthcare provider or women who did not know who the examiner was (*p* = 0.112).

## Discussion

The medical students reported a growing confidence in the clinical situation together with a high satisfaction from the women taking the Pap smear. Moreover, the quality of the Pap smears taken by the medical students was equal to the quality of those taken by the health care staff. Also, the students felt part of the ordinary staff and that they were making a real contribution. All these findings indicate that high patient safety is maintained during this activity, which is of great importance. One might speculate that the education of medical students sometimes is inhibited due to the non-tolerance of endangering patient safety, but this does not seem to be the case in the present study. The concept of the SLC-CCS is unique, considering that most of the practical education in pelvic examination for medical students is teacher-led [19]. The SLC-CCS gave the student the opportunity to develop their professional skills and reflect on their own emotions. Teaching about intimate examinations, such as performing a pelvic examination, to medical students is known to be difficult since many students may feel anxiety about the examination per se [20]. The anxiety may involve fear of hurting the patient, as well as feeling uncomfortable in the intimate situation that the examination represents, touching “the private parts” of another person who is in an awkward and exposed position in relation to the student. However, medical students, at the mandatory course evaluation, recurrently reported a shortage of opportunities to perform a sufficient number of pelvic examinations to master this skill. It seems that more effort must be made by medical schools to accomplish this.

Therefore, the results from this study with SLC-CCS demonstrate that students afforded the opportunity to examine an adequate number of women feel more comfortable in the pelvic examination situation.

The large number of repeated gynecological examinations each student carried out brought with them an embodied awareness and learning experience of variations in normal female anatomy, and this together with the maintained quality of the pap smears and the satisfaction of the women who were examined is a win–win-concept for both for the healthcare system and the students. Since the pelvic examination is performed in all primary health care centers, all physicians need to be comfortable with the examination and the situation per se.

Our results from the questionnaire on the question of whether the women were treated in a respectful and kind manner by the examiner indicated less respectful treatment from the medical students [21], highlighting that it is important to reflect upon “affective subjectivity”, in the context of how examinations are taught to medical students. Reflections of the emotional responses in the students themselves as being care givers and how they will affect work is important to develop a capacity for self-reflection and emotional harmonization with the patients. Ideally, the combination of the patient-led pelvic examination and SLC-CCS would probably give the students even better “affective subjectivity” and thereby improve their ability to treat the patient with respect. In a study by Danielsson et al., 160 medical students responded to a questionnaire about their experiences performing pelvic examinations [22]. The male participants (*n* = 61) experienced having a disadvantage because of their gender, while the female students considered their gender an advantage. Perhaps the gender differences between the group of medical students (ten men) and health care staff (all women) could also explain the small difference in how the women experienced being treated with respect by the examiner since the male gender probably is met with greater resistance in this situation. Another study concluded that students perceived themselves to be more competent performing pelvic examinations compared with staff’s perception of student competency [23]. This opinion may be the same for some of the women attending for a pap smear and may make it harder for the student to achieve a perceived respectful meeting with the women. Overall, the women attending for a Pap smear had a high satisfaction index in all groups, which legitimizes this form of SLC or EPA.

This study has certain strengths and limitations. A strength is the mixed methods model used for evaluation including both qualitative and quantitative data outcome measures including student´s perceptions, medical quality, and women´s experiences. Ideally, the questionnaire should have been distributed during the whole period the SLC-CCS lasted. If so, we would likely have received more answers from women examined by a student. However, we cannot speculate on whether this would have changed the results or if the proportion of answers from the women who did not know who carried out the PAP smear would have been higher or lower. Another shortcoming is that the response rate of the questionnaire could not be estimated. The women either received a paper with a QR code from the person who carried out the test or scanned the QR code, which was available on a poster in the waiting room. This study approach made it impossible to know how many women never scanned the QR code.

## Conclusions

The students expressed a growing confidence in the clinical situation and felt that they were making a real contribution. When performing many gynecological examinations, they experienced that normal female anatomy varied extensively. This, together with a high satisfaction from the women attending the cervical cancer screening program and the fact that the quality of the Pap smears taken by the medical students was equal to the quality of those taken by the health care staff, support the recommendation to include SLC-CCS as part of the medical training.

## Supplementary Information

**Additional file 1. **Information to students about the student-led clinic for cervical cancer screening.

**Additional file 2. **Interview guide.

**Additional file 3. **Questionnaire on women’s experience of the visit.

## Data Availability

The datasets generated and/or analyzed during the current study are available from the corresponding author on reasonable request.

## Abbreviations

EPA: Entrustable professional activity
Pap: Papanicolaou
SLC: Student-led clinics
SLC-CCS: Student led clinic cervical cancer screening
QR: Quick response
