# Supplementary material for: Student-led clinic cervical cancer screening—medical students’ views on progression of learning, quality of Pap smears and women´s experiences of the visit – a mixed methods study
Source: BMC Med Educ. 2023 Apr 5;23:218. doi: 10.1186/s12909-023-04162-y (PMC10077664; doi:10.1186/s12909-023-04162-y)
Supplement: Supplementary file 1 — Additional file 1. Information to students about the student-led clinic for cervical cancer screening. [file 12909_2023_4162_MOESM1_ESM.pdf]

Fifth-year medical students SLC-CCS preparations

On the first day of the term, during roll call, students were given information about SLC-CCS. They were informed that this was a mandatory component of their education and was therefore not optional unless they had special grounds.

On their first day of PLI (placement internship) at the obstetrics and gynaecology departments, students were given an introduction to the theoretical aspects of a gynaecological examination and were also given the opportunity to see and hold the various instruments used for gynaecological examinations and the sampling kits. They were also shown a typical examination room at the clinic, and were shown how the gynaecology chair and lamp are set up etc.

During their introduction, students were also able to watch a pre-recorded video demonstrating in detail a complete gynaecological examination in a simple and educational manner. The film is approximately 40 minutes long, is under copyright and cannot be shared.

A morning or afternoon (four hours) were then allocated for "self-training" for SLC-CCS. The students were given a document which contained practical information and guidelines for preparation and training for SLC-CCS (see below, **Student-led cervical screening clinic**). There were links to web pages containing training material/educational resources regarding cervical screening, human papillomavirus (HPV) and a test that students must have passed before SLC-CCS. This was in order for students not only to be able to personally perform the smear test but to also be able to answer simple questions if the patients had any. No verification was made to ensure that the students performed this training, since we assumed that the students would take responsibility to carry out mandatory components of their medical training.

Student-led cervical screening clinic, Kvinnohälsan Linköping and Motala during placement internship (PLI)

Who: Students in Term 11 (T11) and Course 10 (K10) of the Medical Programme.

Where: Premises on level 5 at Kvinnohälsan (KH), Linköping, as well as the Women's Clinic (Kvinnokliniken) in Motala, one day a week. Two rooms per location and occasion.

Start: spring term, 2021

Student clinic
One or two half-day sessions for each student. Two students per session. Students each work in their own room but should also be available to assist their fellow students before receiving their next patient. If a student is sick, they should be replaced primarily with another student from K10/T11. Two students are counted as one regular (for the invitations). Start is week 5, spring term 21.

Training:
Theoretical training
One half day is added during PLI
Read KK US memorandum: “Cervixcancerprevention”

Review the training material on the RCC website:
https://www.cancercentrum.se/samverkan/vara-uppdrag/prevention-och-tidig-upptackt/gynekologisk-cellprovskontroll/utbildningsmaterial/

HPV communication support:

https://www.cancercentrum.se/globalassets/vara-uppdrag/prevention-tidig-upptackt/gynekologisk-cellprovskontroll/stod-vid-information/kommunikationsstod-for-halso-och-sjukvardspersonal-om-humant-papillomvirus-hpv-181121-nacx.1.pdf

Approved result on the web-based training:
https://webbutbildning-gck.cancercentrum.se/

Practical instruction
See the document entitled *Practical Student Information for Student-Led Cervical Screening*
The two first samples are taken together with a midwife

Guide: Midwife/nurse at KH Linköping and the Women's Clinic in Motala. Samples taken by regular staff in room 3 at Kvinnohälsan and in the designated room in Motala.

Possible advantages:
Students get to practice many gynaecological examinations and patient inspinteractions
Students become more confident with performing gynaecological examinations (useful for future professional practice; i.e., gynaecological examination becomes part of their skill set)
Addresses an area that is included in the objectives for the term but that is covered only to a limited extent.
IPL component
Need for cervical screening not covered.

Evaluation:

- Interview students (15-20 in total). This is done after each gynaecological placement internship in four-week blocks.
- Quality control the number of cervical cells in the sample, and compare the samples taken by healthcare staff with those of the students.
- The women are asked customary simple questions about patient satisfaction following their visit.

Roadmap: If this pilot project is a success for the students and the women involved, with a good sample quality, a student-led cervical screening clinic will be implemented during PLI in the rest of the region and will also include midwifery education.

Steering committee: MB, Caroline Lilliecreutz, MLA Kvinnohälsan, Anna Clara Spetz Holm, chief physician, Josefin Ekholm, healthcare administration, Amanda Zetterström, student representative, Sanna Hansson, student representative, Madeleine Abrandt Dahlgren, professor of medical education.
