# Supplementary material for: Student-led clinic cervical cancer screening—medical students’ views on progression of learning, quality of Pap smears and women´s experiences of the visit – a mixed methods study
Source: BMC Med Educ. 2023 Apr 5;23:218. doi: 10.1186/s12909-023-04162-y (PMC10077664; doi:10.1186/s12909-023-04162-y)
Supplement: Supplementary file 2 — Additional file 2. Interview guide. [file 12909_2023_4162_MOESM2_ESM.pdf]

Introduction

You have recently completed a clinical placement where you also had to be responsible for a student-led clinic for cervical cancer screening (SLC-CCS). Since this student-led clinic is a completely new part of the medical education program, it is important for us to understand if and if so how it supports learning during placements in gynecology.

Topics to be discussed

Attitudes towards gynecological examination/preparation and expectations before SLC-CCS

How did you feel before you got there?
What help did you have from the preparation material you had access to before SLC-CCS?
How prepared did you feel?
What were your expectations?
Was there anything that you thought would be particularly difficult?
think What did you think you would learn?

The meeting with patients during SLC-CCS/conducting gynecological examination

What was it like to meet the patients?
What was it like to conduct the examination?
Something that was difficult?
What did you learn?
What would you have needed to learn more about?

Organization and cooperation

How did you feel received by the staff?
How did support and tutoring work for you?
How could you students collaborate and help each other?
If I say “SLC-CCS provides the opportunity for responsibility and independence during the placement”, what do you say?

Continued development

How does SLC-CCS differ from a 'regular' placement?
What advice would you give to junior student colleagues who are going to do SLC-CCS?
What advice would you give to faculty and staff for the next SLC-CCS?
How has SLC-CCS affected your sense of security in conducting gyn examination?
