# Supplementary material for: Student-led clinic cervical cancer screening—medical students’ views on progression of learning, quality of Pap smears and women´s experiences of the visit – a mixed methods study
Source: BMC Med Educ. 2023 Apr 5;23:218. doi: 10.1186/s12909-023-04162-y (PMC10077664; doi:10.1186/s12909-023-04162-y)
Supplement: Supplementary file 3 — Additional file 3. Questionnaire on women’s experience of the visit. [file 12909_2023_4162_MOESM3_ESM.pdf]

Cervical screening

You have recently had a cervical smear test at Kvinnohälsan. We are interested to know how you feel about the procedure.
We would appreciate it if you could share your views and thoughts by participating in a survey. Responding to the survey is completely voluntary. The answers will be compiled in such a way that individual responses cannot be identified. It is the overall feedback that is of interest, and the responses will be used to improve our work in the future.
The survey takes about five minutes to complete. After filling in the survey form, please leave it in the box labelled "Patientenkäter" *(Patient Surveys).*

---

1. Were you given enough information about the cervical smear test? (either in your invitation, online, from brochures provided or from your healthcare provider)
1 ☐ Yes, absolutely
2 ☐ Partially
3 ☐ No
4 ☐ Does not apply

2. Did it feel like you had to wait a long time in the waiting room before you could have your procedure?
1 ☐ No, I did not have to wait long
2 ☐ Yes, a little too long
3 ☐ Yes, much too long

3. When asking your provider something that was important to you, did you receive answers that you understood?
1 ☐ Yes, absolutely
2 ☐ Partially
3 ☐ No
4 ☐ I didn't dare to ask
5 ☐ I didn't need to ask anything

4. Did you feel that you were treated in a respectful and kind manner in connection with your cervical smear test?
1 ☐ Yes, absolutely
2 ☐ Partially
3 ☐ No

5. As a whole, how would you rate your cervical smear test appointment?
1 ☐ Excellent
2 ☐ Very good
3 ☐ Good
4 ☐ Tolerable
5 ☐ Poor

6. How would you rate your health, in general?
1 ☐ Excellent
2 ☐ Very good
3 ☐ Good
4 ☐ Tolerable
5 ☐ Bad

7. What year were you born in?
(Four figures, e.g., 1945)

____

8. Is Swedish your mother tongue?
1 ☐ Yes
2 ☐ No

9. What is your highest completed education?
1 ☐ Compulsory school or the equivalent
2 ☐ Upper secondary school or the equivalent
3 ☐ University or the equivalent

10. Who performed your cervical smear test
(multiple options possible)
1 ☐ Midwife
2 ☐ Nurse
3 ☐ Assistant Nurse
4 ☐ Medical student
5 ☐ Don't know

11. If there is anything you would like to say that we haven't asked about, we will be happy to read your comments below.

____

____

____

Thank you for taking time to complete this survey and helping us improve!

/The management of Kvinnohälsan in Region Östergötland

[Document::Title] [Document::Number] - [Document::Version]

Postal address	Telephone No.	Fax	Email address
Region Östergötland<br>581 91 Linköping	010-103 00 00<br>(switchboard)	010-103 71 00	region@regionostergotland.se
